# Effects of loading positions on the activation of trunk and hip muscles during flywheel and dumbbell single-leg Romanian deadlift exercises

**DOI:** 10.3389/fphys.2023.1264604

**Published:** 2023-11-29

**Authors:** Ryan Chun Yin Mo, Derrick Chung Wang Ngai, Chapman Cheuk Man Ng, Kenson Ho Sang Sin, Jim Tze Chung Luk, Indy Man Kit Ho

**Affiliations:** ^1^ Department of Sports and Recreation, Technological and Higher Education Institute of Hong Kong (THEi), Chai Wan, Hong Kong SAR, China; ^2^ Chengdu University of Traditional Chinese Medicine, Chengdu, China; ^3^ Asian Academy for Sports and Fitness Professionals (AASFP), Hong Kong, China

**Keywords:** eccentric training, EMG, strength training, gluteus medius, hamstring, gluteus maximus

## Abstract

**Objective:** The study compared the activities of the surface electromyography (sEMG) of trunk and hip muscles during single-leg Romanian deadlift (SLRDL) exercises using a flywheel and dumbbell with different loading positions (ipsilateral and contralateral).

**Method:** Twelve active male subjects with at least 2 years of strength training experience (age: 26.7 ± 3.3 years; weight: 73.9 ± 6.2 kg) participated in this study. sEMG in the percentage of maximum voluntary isometric contraction of four SLRDL exercises (ipsilateral and contralateral loading position for dumbbell and flywheel) in a randomized order for superior gluteus maximus (SGM), inferior gluteus maximus (IGM), gluteus medius (GM), biceps femoris (BF), erector spinae (ES), external oblique (EO), and adductor longus (AL) were measured. One-way repeated measure ANOVA with Bonferroni adjustment (statistical significance at 0.05) and the non-clinical magnitude-based decision with a standardized difference were performed for statistical analysis.

**Results:** The overall results demonstrated a very high level of SGM (105.4%–168.6%) and BF (69.6%–122.4%) muscle activities. A significant moderate increase of sEMG signals in GM, IGM, and ES (dominant side) and a large increase in SGM activity during concentric action when the loading position of flywheel SLRDL was changed from ipsilateral to the contralateral side. No significant difference was observed between flywheel and dumbbell SLRDL exercises.

**Conclusion:** Strength coaches may adopt dumbbell or flywheel SLRDL exercises using the contralateral loading position to simultaneously strengthen the hip extensors and trunk stabilizers effectively.

## 1 Introduction

Romanian deadlift (RDL) is a multi-joint closed-kinetic chain exercise for strengthening the lower limb muscles including hamstring and gluteus maximus (Koderi et al., 2020), back extensors, and also the lumbar region (Mayer et al., 2008). It is well known for its potential benefits in reducing the risk of hamstring injuries (Brughelli and Cronin, 2008). Due to the similar biomechanics with certain phases of weightlifting techniques such as clean and snatch, it can be regarded as a fundamental strengthening variant to improve hip strength, power output, and lifting posture (Weaver and Kerksick, 2017).

Single-leg RDL (SLRDL) is the progression of the RDL taking the benefits from unilateral training. In this regard, Kuki et al. (2018) highlighted its potential effects on boosting neuromuscular activation, especially for the hamstrings and gluteus medius. Apart from the posterior chain musculature, SLRDL also emphasizes the lumbopelvic muscles (Weaver and Kerksick, 2017). Since the unilateral stance limits the base of support increasing instability and the challenge of controlling the center of gravity, greater recruitment of trunk and pelvic stabilizers are required for overcoming the destabilizing torque of the movement (Saeterbakken and Fimland, 2012; Marchetti et al., 2018). Moreover, during SLRDL, the biceps femoris has dual roles including the agonist producing hip extension movement similar to bilateral RDL, and also a knee joint stabilizer in closed kinetic chain exercise (Marchetti et al., 2016). When compared with bilateral exercise, unilateral training was shown to provide a greater total volume of lifting load lifted (Costa et al., 2015) and a cross-education effect in musculoskeletal and neurological rehabilitation (Farthing and Zehr, 2014). Therefore, it is not surprising that previous studies have shown its potential superiority in enhancing strength and sports performance (Kuruganti and Murphy, 2008; Rejc et al., 2010).

In addition to the high hamstring involvement in performing RDL exercises (McAllister et al., 2014), it shares similar biomechanics with the Nordic hamstring curl that both exercises promote an increasing load when the hamstring is fully lengthened (Ribeiro-Alvares et al., 2018). Therefore, it is believed that RDL is also an effective drill to maximize eccentric hamstring strength and reduce the risk of hamstring strain during acceleration and sprinting (Mjølsnes et al., 2004). Recently, a novel training method, accentuated eccentric loading, using a flywheel device was highly promoted and widely studied for its potential superiority in eccentric strength enhancement (Wagle et al., 2017).

Accentuated eccentric loading (AEL) was proposed to overload the eccentric phase to even beyond the maximum magnitude of the concentric load with minimal disturbance to natural mechanics (Wagle et al., 2017). Flywheel, also known as isoinertial training, is one of the AEL training examples applying a linear resistance from a rotating disc with a decent mass attached to the tether keeping a distance from the axis of rotation (Chiu and Salem, 2006). The flywheel torque is based on the radius of gyration, angular acceleration, and mass meanwhile it allows for maximum concentric contraction throughout the full range of motion, an increase of eccentric load, and maximum exertion during the first concentric phase (Tesch et al., 2004; Norrbrand, 2008). Thus, flywheel training was shown to be effective to enhance maximal strength, power, muscular hypertrophy, and functional abilities in vertical and horizontal planes (Petré et al., 2018). Furthermore, some recent studies showed that isoinertial training may evoke higher electromyography (EMG) activities than gravity-dependent weight training (Norrbrand et al., 2011; Núñez et al., 2017). Despite the proposed potential benefits of AEL using flywheel over the traditional free weight training, there is no study comparing the muscle activities between the flywheel and free weight SLRDL exercises.

Although gravity contributes part of the equation in calculating the total resistance during flywheel RDL exercises, the speed and loading of the eccentric phase mostly result from the concentric effort leading to the change of direction of the wheel. Conversely, free weight is based on gravity to induce a stimulus on the musculoskeletal system (Chiu and Salem, 2006). From the biomechanics perspective, when performing the initial downward phase of SLRDL, there will be a relatively lower resistance and hence the demand on muscle activation of both agonists and stabilizers. On the other hand, Tesch et al. (2004) have shown the peak eccentric torque occurred right after the mid-point of the eccentric phase during flywheel knee extension exercise (YoYo™). In this regard, it is believed that the torque and actual resistance applied on the hip and knee joint during free weight and flywheel SLRDL are different.

Besides the sagittal loading applied to the hamstrings for strength enhancement, it is worth noting that aberrant pelvic motion is one of the key factors contributing to hamstring tear (Chumanov et al., 2007). Numerous studies showed that sufficient abdominal oblique activation and lumbopelvic stability are also critical in reducing the risk of hamstring injuries (McCall et al., 2014; Donaldson et al., 2015). Therefore, unilateral training using SLRDL with asymmetric load is believed to be favorable for addressing hamstrings strength and lumbopelvic stability simultaneously (Willardson, 2007). As changing the loading position, especially in the frontal plane can alter the center of gravity (Stastny et al., 2015), it is speculated that changing the loading position (i.e., contralateral vs ipsilateral) in SLRDL will potentially induce different demands and challenges on lumbopelvic stability and relevant stabilizers. In this regard, our gluteus medius is one of the most important pelvic and knee stabilizers to perform both hip abduction and lateral pelvic tilt. Strong evidence on knee injury prevention and rehabilitation (e.g., anterior cruciate ligament injury and anterior knee pain) emphasizing the high-intensity training on this muscle was well established (Powers, 2010; Stastny et al., 2016). Moreover, the superior fibers of the gluteus maximus have a similar location (more lateral on the pelvis) and orientation (diagonal towards the greater trochanter) with the gluteus medius and therefore, these gluteal muscle portions are highly responsible for the pelvic, hip, and knee stability in both the frontal and transverse plane (Powers, 2010; Ho et al., 2020). Since bodyweight SLRDL was shown to be an effective exercise in activating gluteus medius (56%–58% in terms of maximum voluntary isometric contraction) (Stastny et al., 2016), based on the anatomical and biomechanical characteristics of this muscle, it is hypothesized that the change of loading positions potentially increase the muscle activity on the standing leg when the frontal and transverse stability becomes more challenging in performing the unilaterally loaded SLRDL. With limited research in this regard, it is interesting to compare the muscle activities of the trunk and pelvic muscles with different SLRDL loading positions.

To the best knowledge of the authors, the effect of the loading position on muscle activation during SLRDL using a flywheel and dumbbell has not yet been investigated. Therefore, the purpose of this study is to determine the effect of loading position (contralateral vs ipsilateral) and methods (dumbbell vs flywheel) on the surface electromyography (sEMG) of superior gluteus maximus (SGM), inferior gluteus maximus (IGM), gluteus medius (GM), biceps femoris (BF), erector spinae (ES), external oblique (EO), and adductor longus (AL) on SLRDL.

## 2 Materials and methods

### 2.1 Experimental approach to the problem

Subjects were required to attend one familiarization and another data collection session at least 4 days apart. To generalize the result to trained populations, subjects with at least 2 years of strength training experience were recruited. This within-subject repeated measure study investigated the sEMG activities for the four selected SLRDL variants including flywheel with ipsilateral (FLY-Ipsi) and contralateral (FLY-Con) loading positions, and dumbbell with ipsilateral (DB-Ipsi) and contralateral (DB-Con) loading positions in randomized order. Nine lower limb and trunk muscles were selected for measuring muscle activity including the EO of dominant (EO-D) and non-dominant side (EO-ND), ES of dominant (ES-D), and non-dominant side (ES-ND), AL, GM, SGM, IGM, and BF.

### 2.2 Subjects

Twelve young male subjects with at least 2 years of resistance training experience (age: 26.7 ± 3.3 years; weight: 73.9 ± 6.2 kg; height: 172.6 ± 11.1) volunteered to participate in the present study. All subjects completed the Physical Readiness Questionnaire (PAR-Q) and informed consent form meanwhile the study was approved by the Human Research Committee. Subject exclusion criteria include 1) a history of injury and/or surgery on the spinal region or lower extremity or lower back pain in the past 12 months (Ho et al., 2020); 2) uncertain or potential cardiovascular or respiratory diseases indicated in PAR-Q or past medical history; 3) ≥16.6% body fat measured by body composition analyzer (InBody 720, Biospace South Korea); and 4) unable to perform the four variations of the single-leg RDL correctly or occurrence of pain during the familiarization session. Besides, subjects refrained from vigorous activities or resistance training at least 48 h before the data collection session.

### 2.3 Procedures

During the familiarization session, subjects were instructed on the proper technique of all SLRDL exercises. After that, subjects performed a maximal speed of SLRDL using a flywheel with ipsilateral and contralateral loading positions for six repetitions to determine the movement velocity and dumbbell loading intensity. According to Sabido et al. (Sabido et al., 2017), using a light inertial load of 0.025 kg m^2^ on flywheel devices enables the subjects to generate higher power in both concentric and eccentric actions, and therefore, an inertial setting of 0.025 kg m^2^ was used in this study for intensity estimation. A velocity-based training (VBT) sensor (Push Pro Band 2.0) was adopted to the handle of the flywheel device for testing the movement velocity. The data of the first two repetitions for flywheel acceleration and movement amplitude stabilization were discarded (Sabido et al., 2017; Darjan et al., 2020), while the mean velocity of the last four repetitions during the concentric phase was used. Subsequently, the actual dumbbell loading for testing purposes was determined when the subjects successfully performed six repetitions of SLRDL in DB-Con and DB-Ipsi positions using the highest weight with good balance and technique, and the equivalent concentric velocity (±0.1 m/s) as the FLY-Con and FLY-Ipsi accordingly. The pace of the DB-Con and DB-Ipsi was controlled by a digital metronome. A minimum of 4 minutes of rest was given between trials. The weights of dumbbells used for testing purposes as well as the tempos are shown in Table 1.

**TABLE 1:** The weight and tempo of the dumbbell Romanian deadlift used.

Subject ID	Dumbbell on ipsilateral in kg (tempo in m/s)	Dumbbell on contralateral in kg (tempo in m/s)
1	28.0 (0.96)	28.0 (1.01)
2	22.5 (0.98)	20.0 (0.60)
3	28.0 (1.27)	25.0 (0.97)
4	30.0 (0.65)	30.0 (0.54)
5	22.5 (0.97)	22.5 (0.96)
6	28.0 (0.70)	22.5 (0.66)
7	30.0 (0.83)	30.0 (0.78)
8	30.0 (0.88)	32.0 (0.78)
9	30.0 (0.68)	30.0 (0.64)
10	28.0 (0.72)	30.0 (0.70)
11	30.0 (0.74)	28.0 (0.60)
12	22.5 (0.97)	28.0 (0.80)
Average	27.5 (0.86)	27.2 (0.75)

For the data collection session, muscle activities of EO and ES (at L3 level), and the dominant side of GM, SGM, IGM, BF, and AL were measured using the sEMG system (MyoMuscle, Noraxon, United States, Inc., Scottsdale, AZ) at a sample rate of 1,000 Hz with the TeleMyo DTS Desk Receiver. The sites for electrode placement were rubbed and cleaned with alcohol pads until the skin showed slight redness meanwhile the hair was shaved to avoid skin impedance and maximize the quality of the sEMG signals (Ho et al., 2020). Disposal sEMG electrodes containing silver-silver chloride (Ag/AgCl) and conductive wet gel (Blue Sensor T-00-S, Ambu Inc., Malaysia), with a center-to-center inter-electrode distance of 35 mm were used (Ho et al., 2020). Several 3M™ tapes were applied to secure the electrodes. Raw data were processed with MyoResearch 3.8 software (Noraxon United States, Inc., Scottsdale, AZ) with full-wave rectified, band-pass filtered from 50 to 500 Hz and smoothed via the root-mean-square (RMS) algorithm and 100-millisecond moving window.

Based on the recommendation from previous studies, electrodes for the following muscles were placed: EO) a diagonal line with 45° and upper-level of the anterior superior iliac spine which closed by the level of the umbilicus; ES) 3 cm lateral to the spinous process and nearly level with the iliac crest between L3 and L4 vertebrae (Escamilla et al., 2010); SGM) upper and laterally to the middle of the line connecting from the posterior superior iliac spine (PSIS) and the posterior greater trochanter; IGM) inferiorly and medially to the middle of the line connecting from the PSIS and the posterior greater trochanter, and approximately 2.5–5 cm above the gluteal fold (Selkowitz et al., 2016); GM) a half of the distance between the iliac crest and the greater trochanter anteriorly and upper from the gluteus maximus; AL) medially to the thigh and same to the proximal one-third of the distance from the pubic tubercle to the linea aspera on the femur (Serner et al., 2014); BF) a half of the line between ischial tuberosity and lateral epicondyle (Ho et al., 2020). The dominant side of the body was determined by the usual kicking leg. All electrodes were placed parallel to the muscle fibers for better sensitivity.

### 2.4 Data collection and normalization

Subjects initially performed a 5-min self-paced slow jogging on the treadmill as the standardized warm-up before performing SLRDLs. After that, subjects performed the MVIC tests for EMG normalization. The MVIC testing protocols of selected muscles were referenced from the positions described in Kendall et al. (2005), Escamilla et al. (2010), Selkowitz et al. (2016), and Delmore et al. (2014). The peak EMG amplitude of each muscle was obtained through three maximal isometric contractions of 5 seconds using manual resistance with 1-min rest between trials (Burden, 2010; Chuang and Acker, 2019). Verbal encouragement was consistently provided during the MVIC test. After that, subjects performed four SLRDL variants in a randomized order for one set of six repetitions with a 4 minutes rest between exercises (Pearson et al., 2000). Both concentric and eccentric actions in all SLRDL exercises were performed with maximal speed. Before data collection, the foot placement and the grip position of SLRDL exercises were standardized. Throughout the trials, subjects maintained the knee flexion angle at approximately 15°, and at the bottom position upon the end of the eccentric phase, the trunk position was about parallel to the ground. If subjects failed to complete the six repetitions continuously, they were required to redo after a 4-min rest until a successful trial was made. The last 4 repetitions of each SLRDL variation were used for further analysis. For the FLY-Con and FLY-Ipsi conditions, subjects started with a unilateral stance with the dominant leg right behind the handle of the flywheel device such that the shoulder was in line with the handle. Meanwhile, the instructor slowly rotated the wheel of the flywheel device to lower the grip and prepare for the initiation. Once the trunk paralleled the ground, the subjects were instructed to fully extend the hip to initiate the SLRDL exercise. Subjects were encouraged to give maximal speed and effort throughout the flywheel SLRDL exercises. For the FLY-Con, the handle was gripped by the non-dominant side and in line with the contralateral shoulder. Subjects performed the DB-Con and DB-Ipsi conditions with identical body postures as the FLY-Con and FLY-Ipsi respectively.

### 2.5 Statistical analysis

sEMG data were normalized as the percentage of MVIC (%MVIC) and expressed as mean and ±SD. The activation was deemed low, moderate, high, and very high for 0%–20%, 21%–40%, 41%–60%, and over 60% respectively (Escamilla et al., 2009). The data of the dominant side was defined as the usual limb for kicking a ball (Selkowitz et al., 2016). A two-way mixed model intraclass correlation coefficient (ICC) was used for analyzing the relative test-retest reliability throughout the final four repetitions. One-way repeated measure ANOVA was used for analyzing the difference in muscle activity between SLRDL variations. A *post hoc* pairwise comparison with Bonferroni adjustment was applied while 0.05 was set for statistical significance.

In addition to the traditional statistical analyses, the non-clinical magnitude-based decision and precision of estimation were used with differences between conditions assessed via corresponding 90% confidence intervals, and their standardized effect (mean difference divided by the standardized unit) of each pairwise comparison was calculated. The smallest worthwhile difference was set at 0.2 while thresholds for the magnitudes of effects were: 0.2, small; 0.6, moderate; 1.2, large; 2.0, very large; and 4.0, extremely large (Hopkins et al., 2009). The effects were unclear if the respective 90% confidence intervals crossed the thresholds of the effect being substantially positive and negative by >5%. Otherwise, the clear effect with the percentage likelihood of effects being substantially positive, trivial, and substantially negative was observed, and the corresponding qualitative inference was produced. The probabilistic terms for classifying likelihood values were as follows: <0.5%, almost certainly not; 0.5%–5% very unlikely; 5%–25% unlikely; 25%–75% possibly; 75%–95% likely; 95%–99.5% very likely; >99.5% almost certainly (Hopkins et al., 2009). All data analyses were performed using RStudio software (version 1.2.5001).

## 3 Results

**FIGURE 1:**
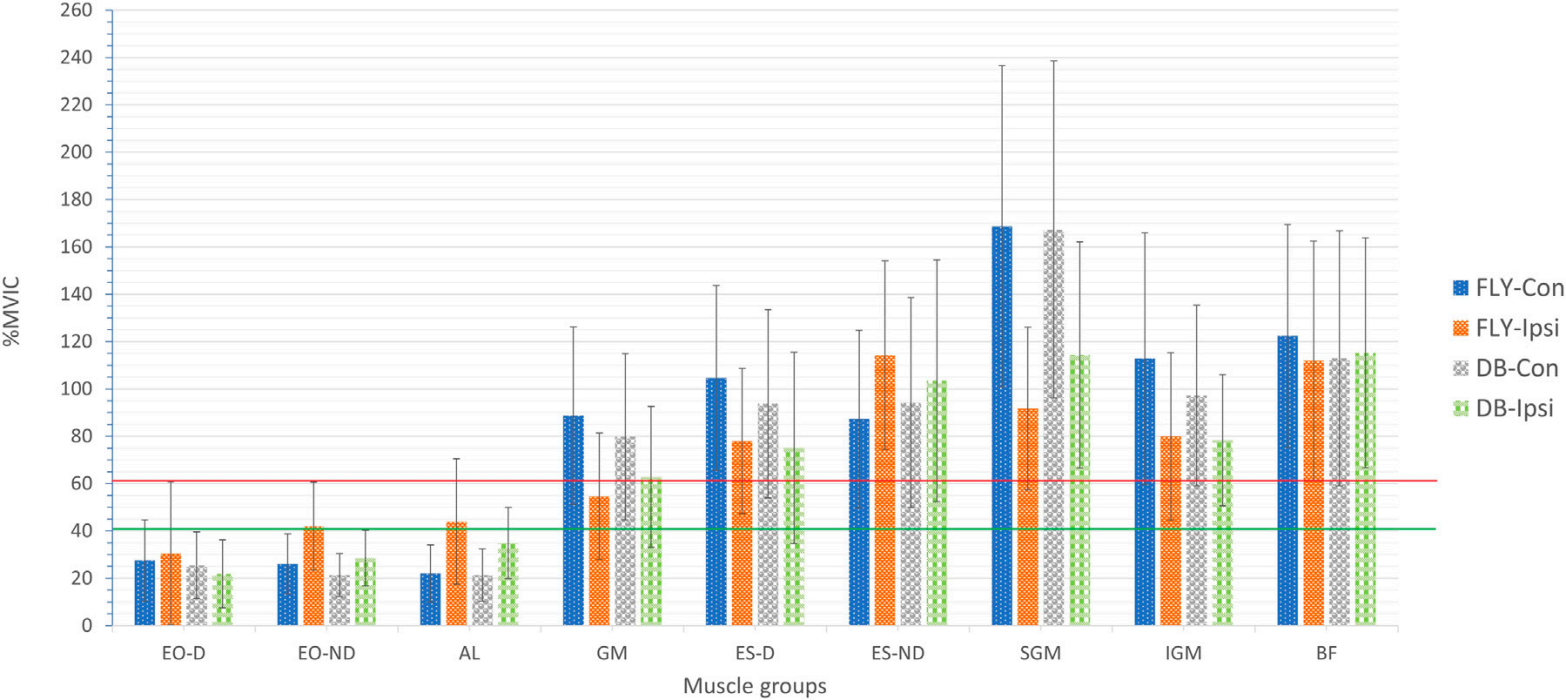
The results of magnitude-based decision for biceps femoris and adductor longus during the concentric phase AL: adductor longus; BF: biceps femoris; FLY-Con: flywheel with contralateral loading; FLY-Ipsi: flywheel with ipsilateral loading; DB-Con: dumbbell with contralateral loading; DB-Ipsi: dumbbell with ipsilateral loading.

**FIGURE 2:**
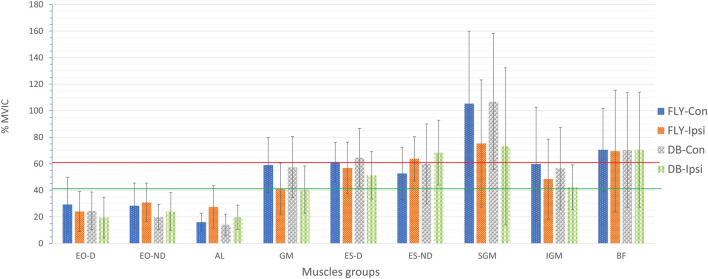
The results of magnitude-based decision for erector spinae muscle during the eccentric phase ES-D: the dominant side of erector spinae; ES-ND: the non-dominant side of erector spinae; FLY-Con: flywheel with contralateral loading; FLY-Ipsi: flywheel with ipsilateral loading; DB-Con: dumbbell with contralateral loading; DB-Ipsi: dumbbell with ipsilateral loading.

The muscle activities in terms of %MVIC of all conditions were presented in Table 2 and graphically in Figure 1. All data were normally distributed based on the Shapiro-Wilk test (*p* > 0.05) or visual inspection, and most sEMG results demonstrated good to excellent relative test-retest reliability (ICCs: 0.75–0.99) except the EO-D in FLY-Con during the concentric phase (ICC = 0.49) and ES-D in FLY-Ipsi condition during the eccentric phase (ICC = 0.43) providing poor reliability. Regarding the magnitude of the sEMG signals, the concentric (FYL-Con: 168.6% ± 67.9%; DB-Con: 167.2% ± 71.2%) and eccentric (FYL-Con: 105.4% ± 54.6%; DB-Con: 106.8% ± 51.3%) contraction of SGMax demonstrated a very high level of activation (>60%) (Figures 1, 2). BF muscle also produced very high muscle activities in all conditions (69.6%–122.4%) (Table 2). Conversely, both concentric and eccentric phases of EO-D only yielded low to moderate levels of activation in all conditions.

**TABLE 2:** sEMG values of the trunk and lower limb muscles of the 4 variations of single-leg Romanian deadlift (mean ± SD).

	EO-D	EO-ND	AL	GM	ES-D	ES-ND	SGM	IGM	BF
**Concentric phase**									
FLY-Con	27.49 ± 17.15	26.10 ± 12.72	22.00 ± 12.05	88.67 ± 37.46	104.63 ± 39.05	87.26 ± 37.45	168.62 ± 67.93	112.8 ± 53.11	122.40 ± 47.08
FLY-Ipsi	30.49 ± 30.29	41.94 ± 18.60	43.91 ± 26.51	54.61 ± 26.73	78.06 ± 30.67	114.25 ± 39.81	91.68 ± 34.30	79.94 ± 35.42	111.92 ± 50.54
DB-Con	25.47 ± 14.11	21.30 ± 9.11	21.35 ± 11.10	79.80 ± 35.06	93.79 ± 39.77	94.27 ± 44.35	167.32 ± 71.18	97.21 ± 38.13	112.96 ± 53.86
DB-Ipsi	21.86 ± 14.36	28.56 ± 11.80	34.90 ± 15.05	62.85 ± 29.71	75.05 ± 40.37	103.47 ± 51.03	114.36 ± 47.81	78.28 ± 27.70	115.22 ± 48.61
**Eccentric phase**									
FLY-Con	29.37 ± 20.53	28.47 ± 17.04	16.07 ± 6.70	59.03 ± 20.81	61.20 ± 15.00	52.81 ± 19.77	105.35 ± 54.59	59.81 ± 42.89	70.61 ± 31.17
FLY-Ipsi	24.03 ± 15.05	30.95 ± 14.61	27.53 ± 15.94	41.22 ± 19.24	56.98 ± 19.25	63.77 ± 16.63	75.26 ± 47.93	48.47 ± 30.13	69.63 ± 45.79
DB-Con	24.63 ± 14.10	20.02 ± 9.46	14.10 ± 8.02	57.49 ± 23.00	64.61 ± 21.96	59.91 ± 30.13	106.80 ± 51.29	56.58 ± 30.78	70.39 ± 43.17
DB-Ipsi	19.52 ± 15.32	24.03 ± 14.24	19.75 ± 9.10	40.59 ± 17.78	51.31 ± 17.82	68.45 ± 24.36	73.11 ± 29.25	42.45 ± 16.78	70.62 ± 43.39

EO-D: the dominant side of external oblique; EO-ND: the non-dominant side of external oblique; AL: adductor longus; GM: gluteus medius; ES-D: the dominant side of erector spinae; ES-ND: the non-dominant side of erector spinae; SGM: superior gluteus maximus; IGM: inferior gluteus maximus; BF: biceps femoris; FLY-Con: flywheel with contralateral loading; FLY-Ipsi: flywheel with ipsilateral loading; DB-Con: dumbbell with contralateral loading; DB-Ipsi: dumbbell with ipsilateral loading.

The sEMG values of most muscles observed in different conditions showed significant differences using one-way repeated measure ANOVAs (*p* < 0.05) except the EO-D and BF muscles in both concentric and eccentric phases, and the ES-D during the eccentric phase showing no significant difference among four training conditions.

When comparing the loading position, *post hoc* and MBD pairwise comparisons showed a significant moderate increase of sEMG activities in GM, IGM, and ES-D and a large increase in SGM activity during concentric action when changing the loading position from FLY-Ipsi to FLY-Con. Similarly, DB-Con showed a significant moderate increase in SGM in concentric action, both GM, ES-D, and SGM during eccentric action over the DB-Ipsi condition (Table 3).

**TABLE 3:** Comparison of sEMG activities between loading positions during the single leg Romanian deadlift exercises.

	FLY-Con - FLY-Ipsi		DB-Con - DB-Ipsi	
	Difference in mean %MVIC±SD (95% CI)	Standardized difference (90% CI)	Difference in mean %MVIC±SD (95% CI)	Standardized difference (90% CI)
**Concentric phase**				
EO-D	−2.99 ± 17.59 (−19.08, 13.10)	−0.11 (−0.45, 0.23)	3.62 ± 9.28 (−4.89, 12.12)	0.24 (−0.07, 0.54)
EO-ND	−15.84 ± 17.41 (−31.93, 0.24)	−0.92 (−1.44, −0.4)	−7.53 ± 11.95 (−18.39, 3.87)	−0.64 (−1.18, −0.09)
AL	−21.90 ± 20.66 (−41.09, −2.71)^a^	−0.98 (−1.47, −0.5)	−13.55 ± 10.27 (−23.05, −4.06)**	−0.95 (−1.32, −0.58)
GM	34.06 ± 18.12 (17.24, 50.88)**	0.97 (0.70, 1.24)	16.95 ± 17.29 (0.99, 32.01)^a^	0.48 (0.23, 0.74)
ES-D	26.57 ± 12.93 (14.90, 38.25)**	0.70 (0.53, 0.87)	18.75 ± 14.88 (4.93, 32.57)**	0.43 (0.25, 0.61)
ES-ND	−26.993 ± 19.14 (−44.78, −9.21)**	−0.65 (−0.88, −.041)	−9.20 ± 23.01 (−30.70, 12.29)	−0.18 (−0.41, 0.05)
SGM	76.943 ± 45.26 (34.92, 118.97)**	1.32 (0.92, 1.73)	52.97 ± 49.5 (7.22, 98.71)^a^	0.81 (0.42, 1.2)
IGM	32.865 ± 23.31 (11.39, 54.34)**	0.67 (0.43, 0.92)	18.93 ± 19.52 (0.83, 37.04)^a^	0.53 (0.24, 0.81)
BF	10.480 ± 25.56 (−13.05, 34.01)	0.2 (−0.05, 0.45)	−2.27 ± 20.59 (−22.24, 17.71)	−0.04 (−0.24, 0.16)
**Eccentric phase**				
EO-D	5.39 ± 17.22 (−10.42, 21.10)	0.27 (−0.18, 0.73)	5.12 ± 10.88 (−5.05, 15.28)	0.32 (−0.04, 0.68)
EO-ND	−2.48 ± 6.71 (−8.75, 3.79)	−0.14 (−0.35, 0.06)	−4.01 ± 11.47 (−14.52, 6.49)	−0.31 (−0.76, 0.14)
AL	−11.46 ± 13.17 (−23.61, 0.6)	−0.87 (−1.38, −0.35)	−5.65 ± 4.95 (−10.24, −1.06)^a^	−0.61 (−0.89, −0.33)
GM	17.81 ± 13.49 (5.28, 30.34)**	0.82 (0.50, 1.15)	16.90 ± 12.24 (5.57, 28.23)**	0.76 (0.47, 1.05)
ES-D	4.22 ± 18.34 (−13.02, 21.46)	0.23 (−0.29, 0.74)	13.30 ± 8.42 (5.52, 21.08)**	0.62 (0.41, 0.82)
ES-ND	−10.96 ± 10.54 (−20.69, −1.23)^a^	−0.55 (−0.83, −0.28)	−8.54 ± 13.56 (−21.01, 3.92)	−0.29 (−0.52, −0.05)
SGM	30.08 ± 25.07 (6.94, 53.23)**	0.54 (0.31, 0.77)	33.69 ± 28.55 (7.22, 60.16)^a^	0.75 (0.42, 1.07)
IGM	11.33 ± 18.89 (−6.13, 28.79)	0.28 (0.04, 0.53)	14.12 ± 16.23 (−0.90, 29.15)	0.53 (0.21, 0.84)
BF	0.98 ± 32.77 (−32.23, 34.19)	0.02 (−0.42, 0.46)	−0.22 ± 22.3 (−12.68, 12.24)	−0.01 (−0.18, 0.17)

EO-D: the dominant side of external oblique; EO-ND: the non-dominant side of external oblique; AL: adductor longus; GM: gluteus medius; ES-D: the dominant side of erector spinae; ES-ND: the non-dominant side of erector spinae; SGM: superior gluteus maximus; IGM: inferior gluteus maximus; BF: biceps femoris; FLY-Con: flywheel with contralateral loading; FLY-Ipsi: flywheel with ipsilateral loading; DB-Con: dumbbell with contralateral loading; DB-Ipsi: dumbbell with ipsilateral loading; CI: confidence interval.

^a^ Significant difference with *p* < 0.05; **: significant difference with *p* < 0.01.

**FIGURE 3:**
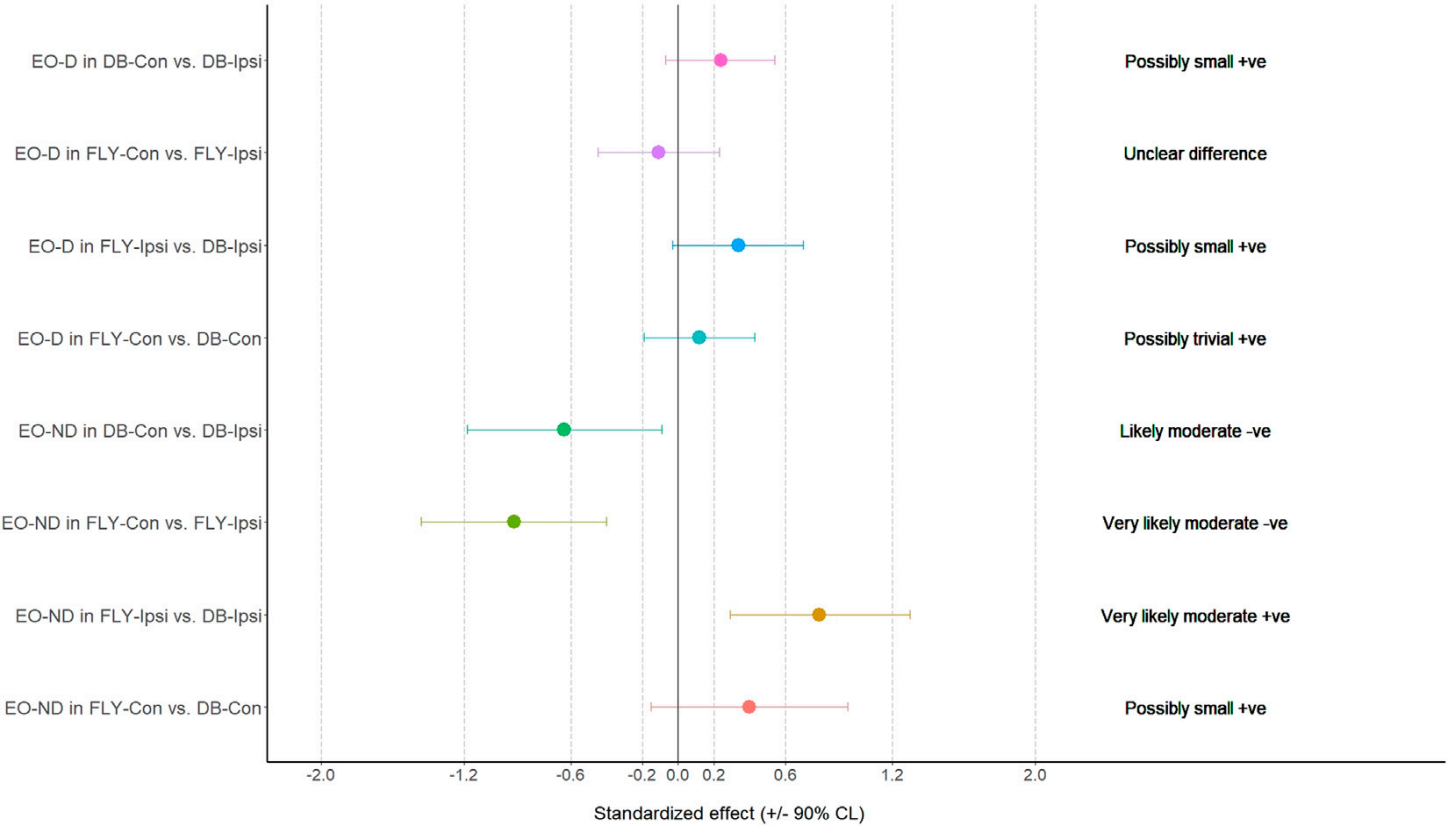
The results of magnitude-based decision for external oblique muscle during the eccentric phase EO-D: the dominant side of external oblique; EO-ND: the non-dominant side of external oblique; FLY-Con: flywheel with contralateral loading; FLY-Ipsi: flywheel with ipsilateral loading; DB-Con: dumbbell with contralateral loading; DB-Ipsi: dumbbell with ipsilateral loading.

**FIGURE 4:**
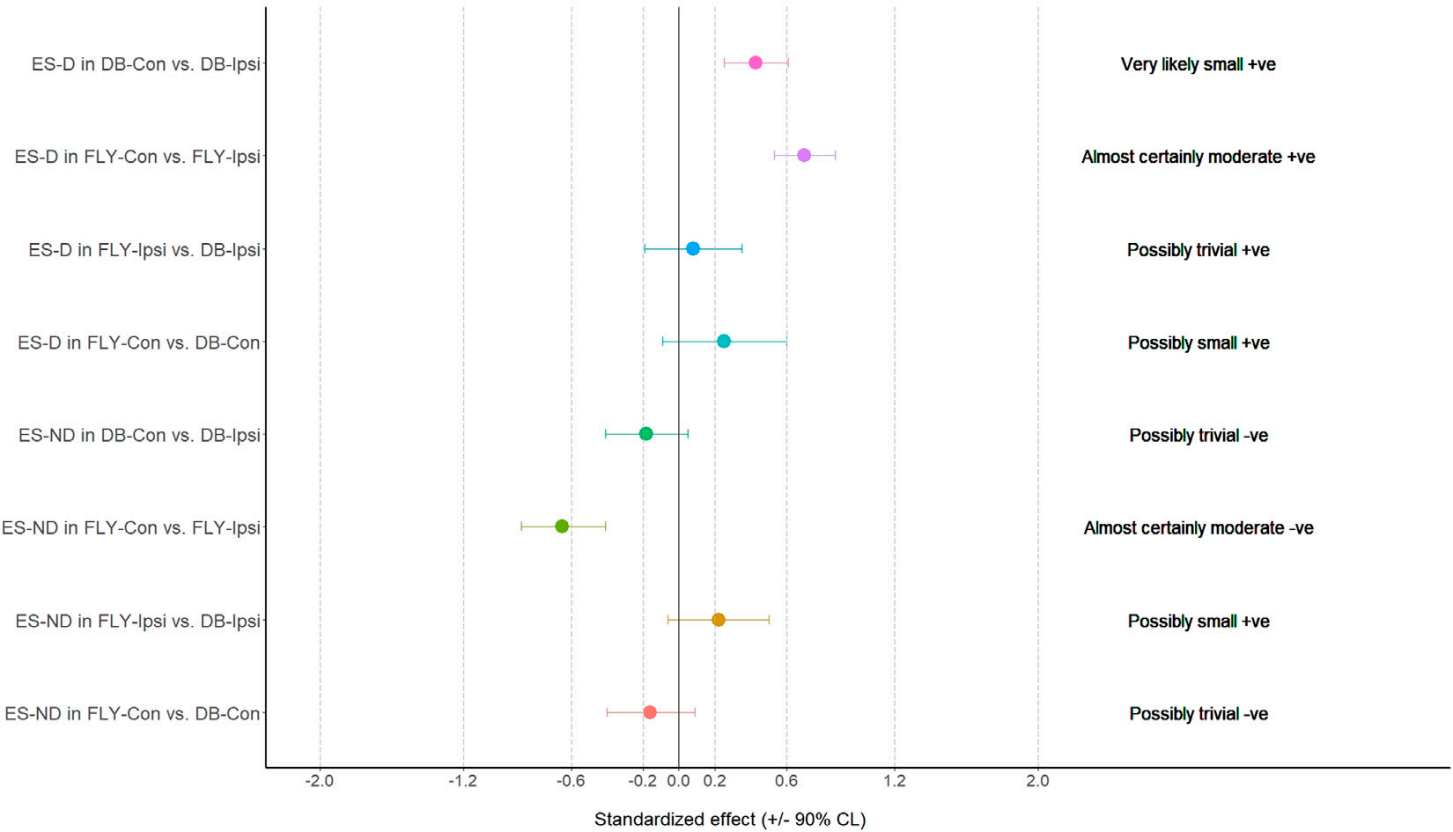
The results of magnitude-based decision for gluteal muscles during the eccentric phase GM: gluteus medius; SGM: superior gluteus maximus; IGM: inferior gluteus maximus; FLY-Con: flywheel with contralateral loading; FLY-Ipsi: flywheel with ipsilateral loading; DB-Con: dumbbell with contralateral loading; DB-Ipsi: dumbbell with ipsilateral loading.

**FIGURE 5:**
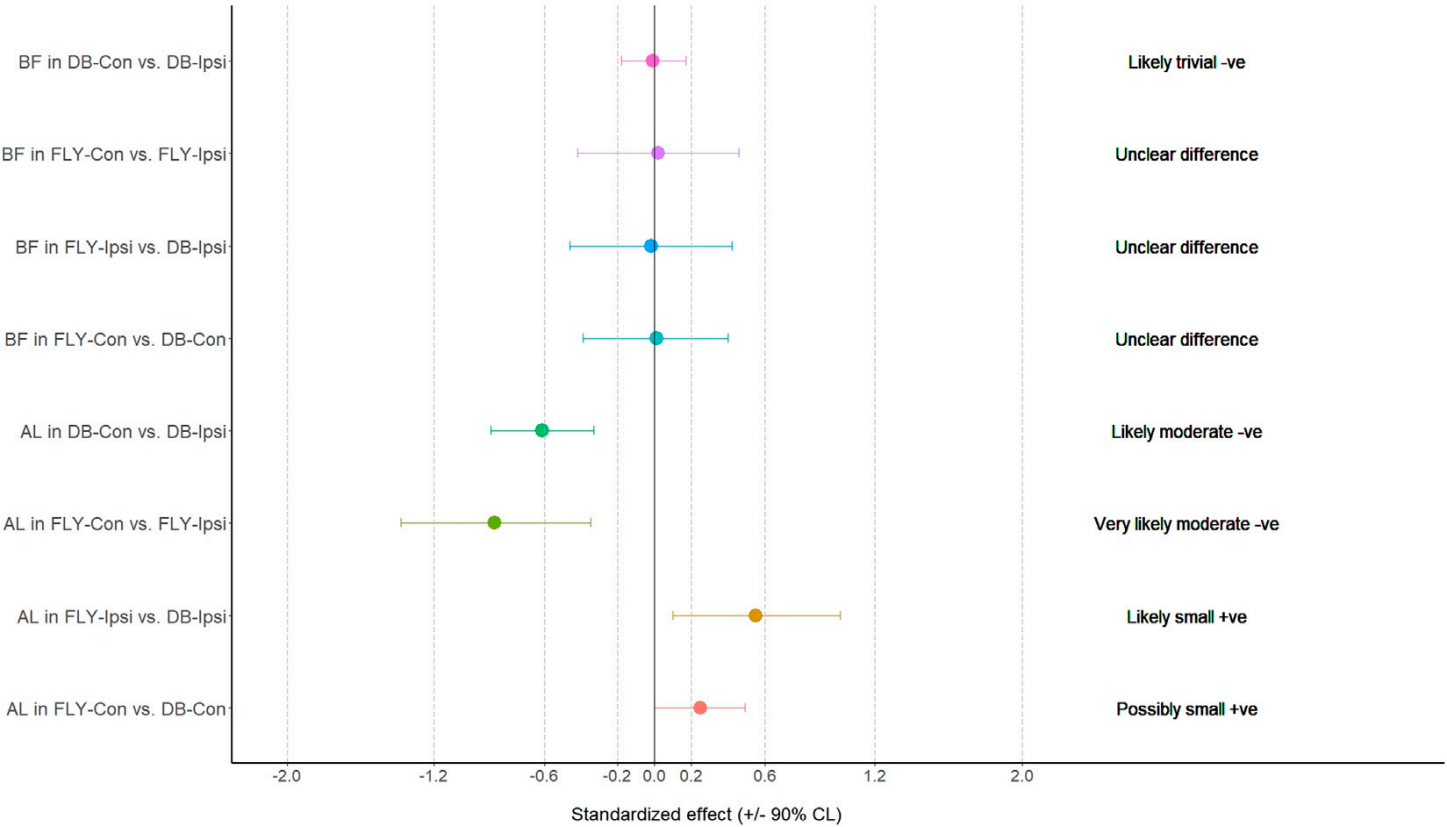
The results of magnitude-based decision for biceps femoris and adductor longus during the eccentric phase AL: adductor longus; BF: biceps femoris; FLY-Con: flywheel with contralateral loading; FLY-Ipsi: flywheel with ipsilateral loading; DB-Con: dumbbell with contralateral loading; DB-Ipsi: dumbbell with ipsilateral loading.

**FIGURE 6:**
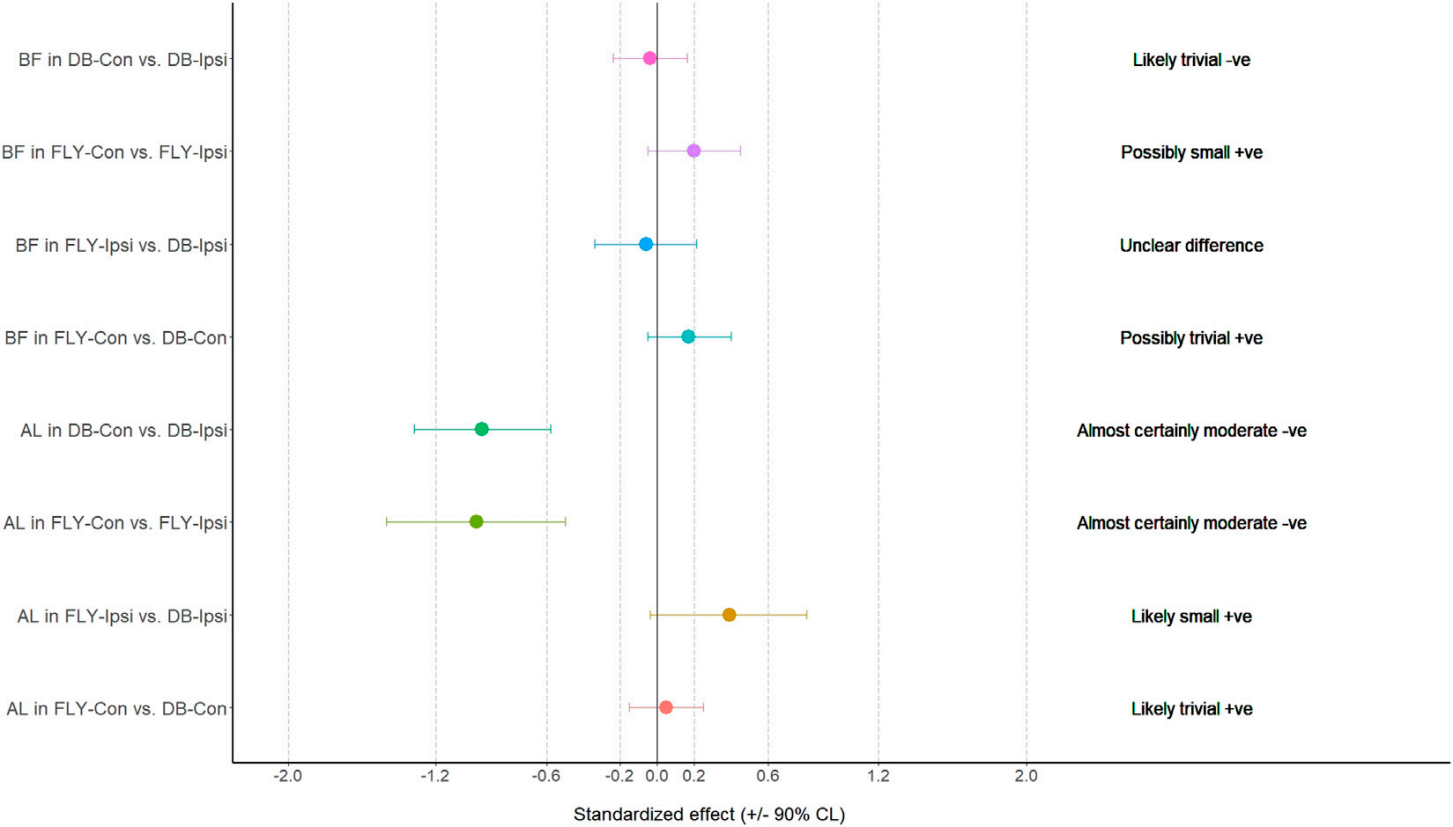
sEMG values of trunk and lower limb muscles of the 4 variations of single-leg Romanian deadlift during concentric phase EO-D: the dominant side of external oblique; EO-ND: the non-dominant side of external oblique; AL: adductor longus; GM: gluteus medius; ES-D: the dominant side of erector spinae; ES-ND: the non-dominant side of erector spinae; SGM: superior gluteus maximus; IGM: inferior gluteus maximus; BF: biceps femoris; FLY-Con: flywheel with contralateral loading; FLY-Ipsi: flywheel with ipsilateral loading; DB-Con: dumbbell with contralateral loading; DB-Ipsi: dumbbell with ipsilateral loading; The red line represents the threshold for effective strengthening effect with a very high muscle activity (>60%) and the green line presents the high muscle activity (>40%).

**FIGURE 7:**
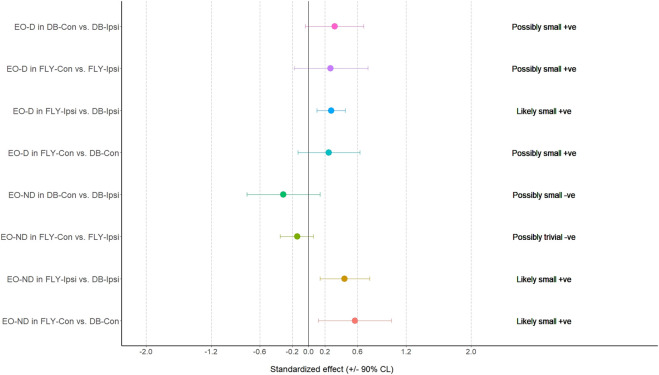
sEMG values of trunk and lower limb muscles of the 4 variations of single-leg Romanian deadlift during eccentric phase EO-D: the dominant side of external oblique; EO-ND: the non-dominant side of external oblique; AL: adductor longus; GM: gluteus medius; ES-D: the dominant side of erector spinae; ES-ND: the non-dominant side of erector spinae; SGM: superior gluteus maximus; IGM: inferior gluteus maximus; BF: biceps femoris; FLY-Con: flywheel with contralateral loading; FLY-Ipsi: flywheel with ipsilateral loading; DB-Con: dumbbell with contralateral loading; DB-Ipsi: dumbbell with ipsilateral loading; The red line represents the threshold for effective strengthening effect with a very high muscle activity (>60%) and the green line presents the high muscle activity (>40%).

**FIGURE 8:**
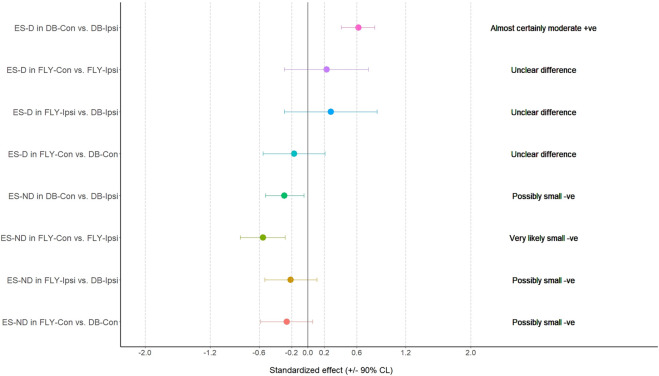
The results of magnitude-based decision for external oblique muscle during the concentric phase EO-D: the dominant side of external oblique; EO-ND: the non-dominant side of external oblique; FLY-Con: flywheel with contralateral loading; FLY-Ipsi: flywheel with ipsilateral loading; DB-Con: dumbbell with contralateral loading; DB-Ipsi: dumbbell with ipsilateral loading.

**FIGURE 9:**
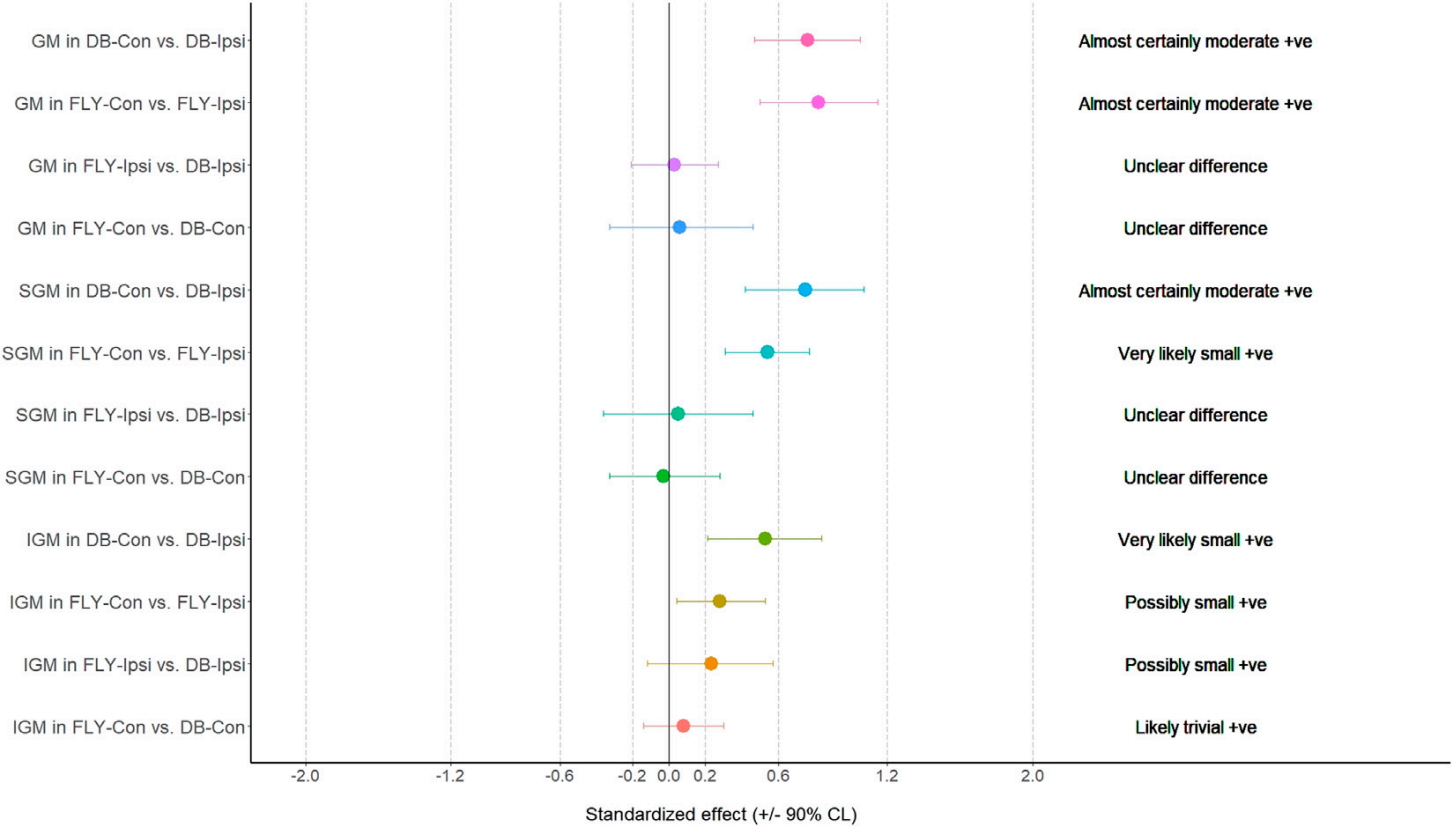
The results of magnitude-based decision for erector spinae muscle during the concentric phase ES-D: the dominant side of erector spinae; ES-ND: the non-dominant side of erector spinae; FLY-Con: flywheel with contralateral loading; FLY-Ipsi: flywheel with ipsilateral loading; DB-Con: dumbbell with contralateral loading; DB-Ipsi: dumbbell with ipsilateral loading.

**FIGURE 10:**
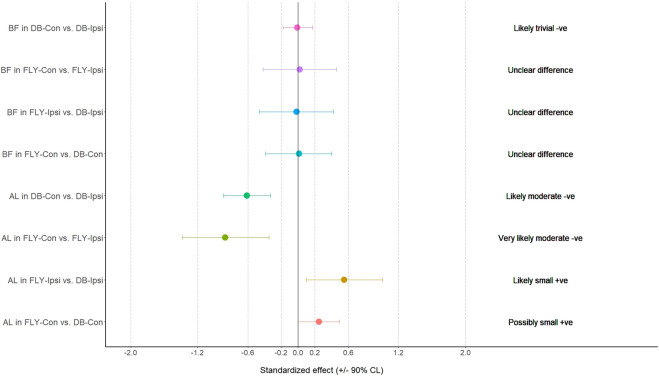
The results of magnitude-based decision for gluteal muscles during the concentric phase GM: gluteus medius; SGM: superior gluteus maximus; IGM: inferior gluteus maximus; FLY-Con: flywheel with contralateral loading; FLY-Ipsi: flywheel with ipsilateral loading; DB-Con: dumbbell with contralateral loading; DB-Ipsi: dumbbell with ipsilateral loading.

When comparing the training methods in the same loading position, no significant difference was observed between FLY and DB conditions while MBD showed a small increase in ES-D, GM, and IGM during concentric action and a small increase in EO-D, EO-ND, and AL in eccentric action when changing from DB-Con to FLY-Con. Similarly, a small increase in EO-D, AL, and ES-ND while a moderate increase in EO-ND during concentric action, and a small increase in EO-D, EO-ND, AL, ES-D, and IGM during the eccentric phase when changing from DB-Ipsi to FLY-Ipsi were observed (Table 4). All MBD results were shown in Figures 3–10.

**TABLE 4:** Comparison of sEMG activities between loading methods (dumbbell vs flywheel) during the single leg Romanian deadlift exercises.

	FLY-Con - DB-Con		FLY-Ipsi - DB-Ipsi	
	Difference in mean %MVIC±SD (95% CI)	Standardized difference (90% CI)	Difference in mean %MVIC±SD (95% CI)	Standardized difference (90% CI)
**Concentric phase**				
EO-D	2.02 ± 10.11 (−7.29, 11.33)	0.12 (−0.19, 0.43)	8.27 ± 17.98 (−8.19, 25.45)	0.34 (−0.03, 0.70)
EO-ND	4.80 ± 12.62 (−6.94, 16.36)	0.4 (−0.15, 0.95)	13.38 ± 16.52 (−1.89, 28.66)	0.79, (0.29, 1.30)
AL	0.65 ± 5.02 (−3.83, 5.13)	0.05 (−0.15, 0.25)	9.00 ± 19.16 (−8.70, 26.70)	0.39 (−0.04, 0.81)
GM	8.87 ± 26.87 (−15.83, 33.56)	0.23 (−0.13, 0.58)	−8.25 ± 4.34 (−22.77, 6.27)	−0.27 (−0.54, 0.00)
ES-D	10.84 ± 28.53 (−15.47, 37.15)	0.25 (−0.09, 0.60)	3.02 ± 20.11 (−15.65, 21.69)	0.08 (−0.19, 0.35)
ES-ND	−7.01 ± 21.24 (−26.43, 12.42)	−0.16 (−0.40, 0.09)	10.78 ± 26.96 (−14.18, 35.75)	0.22 (−0.06, 0.50)
SGM	1.30 ± 64.85 (−51.04, 53.63)	0.02 (−0.37, 0.41)	−22.68 ± 28.71 (−49.16, 3.80)	−0.50 (−0.83, −0.17)
IGM	15.59 ± 22.93 (−5.57, 36.75)	0.31 (0.07, 0.55)	1.66 ± 18.43 (−15.47, 18.79)	0.05 (−0.23, 0.33)
BF	9.45 ± 23.61 (−12.69, 31.59)	0.17 (−0.05, 0.4)	−3.30 ± 27.49 (−29.73, 23.13)	0.17, (−0.95, 0.40)
**Eccentric phase**				
EO-D	4.74 ± 13.95 (−8.29, 17.77)	0.25 (−0.13, 0.63)	4.52 ± 5.51 (−0.60, 9.63)	0.28 (0.10, 0.45)
EO-ND	8.45 ± 12.81 (−3.49, 20.39)	0.57 (0.12, 1.02)	6.92 ± 9.10 (−0.54, 15.37)	0.44 (0.14, 0.75)
AL	1.96 ± 3.78 (−1.51, 5.44)	0.25 (0.00, 0.49)	7.78 ± 12.34 (−3.66, 19.21)	0.55 (0.10, 1.01)
GM	1.54 ± 17.10 (−15.15, 18.23)	0.06 (−0.33, 0.46)	0.63 ± 9.00 (−7.96, 9.22)	0.03 (−0.21, 0.27)
ES-D	−3.42 ± 14.86 (−17.26, 10.42)	−0.17 (−0.55, 0.21)	5.67 ± 21.79 (−16.67, 26.00)	0.28 (−0.29, 0.85)
ES-ND	−7.10 ± 16.90 (−22.91, 8.72)	−0.26 (−0.58, 0.06)	−4.68 ± 14.18 (−17.62, 8.26)	−0.21 (−0.53, 0.11)
SGM	−1.45 ± 36.25 (−32.99, 30.09)	−0.03 (−0.33, 0.28)	2.16 ± 35.95 (−28.97, 33.29)	0.05 (−0.36, 0.46)
IGM	3.23 ± 17.00 (−12.75, 19.21)	0.08 (−0.14, 0.30)	6.02 ± 17.70 (−10.28, 22.32)	0.23 (−0.12, 0.57)
BF	0.22 ± 10.85 (−28.76, 29.13)	0.01 (−0.39, 0.40)	−0.99 ± 49.45 (−38.70, 36.72)	−0.02 (−0.46, 0.42)

EO-D: the dominant side of external oblique; EO-ND: the non-dominant side of external oblique; AL: adductor longus; GM: gluteus medius; ES-D: the dominant side of erector spinae; ES-ND: the non-dominant side of erector spinae; SGM: superior gluteus maximus; IGM: inferior gluteus maximus; BF: biceps femoris; FLY-Con: flywheel with contralateral loading; FLY-Ipsi: flywheel with ipsilateral loading; DB-Con: dumbbell with contralateral loading; DB-Ipsi: dumbbell with ipsilateral loading; CI: confidence interval.

^a^ Significant difference with *p* < 0.05; **: significant difference with *p* < 0.01.

## 4 Discussion

The current study aims to compare the muscle activities of EO-D, EO-ND, AL, GM, ES-D, ES-ND, SGM, IGM, and BF between the four variations of SLRDL (FLY-Con, DB-Con, FLY-Ipsi & DB-Ipsi). According to Macadam et al. (2015), approximately 40%–60% of MVIC is required to produce sufficient stimulus for improving muscle strength and therefore, SLRDL exercises yielded >60% (very high activation) were deemed adequate for strength enhancement in this study.

Regarding the activation of all our selected muscles, SLRDL variations were effective in strengthening the BF, IGM, SGM, ES-D, and ES-ND while FLY variations were also useful for strengthening GM during the concentric phase (Figure 1). For the eccentric action, only BF and SGM were highly activated in all SLRDL conditions to provide sufficient strengthening effect while the DB conditions could also produce good strengthening stimuli to ES-ND (Figure 2). Since the BF and SGM are the major hip extensors, it is not surprised for such high muscle activities in both concentric and eccentric SLRDL actions especially when movements were performed with maximum movement speed potentially favoring the additional recruitment of fast-twitch fibers and higher sEMG signal (Sakamoto and Sinclair, 2011). Recent literature has stated that there were approximately 30%–40% and 15%–20% of MVIC in BF during concentric and eccentric actions respectively using 12 repetition maximum of unilateral barbell RDL exercises, and it was regarded as the second-lowest muscle activity among selected hamstring exercises (e.g., good morning and straight leg bridge) (Hegyi et al., 2018). Conversely, our study produced 112%–123% and 70%–71% of MVIC during the concentric and eccentric phase of DB and FLY SLRDL variations respectively when six repetitions with maximum speed were performed. Similarly, Koderi et al. (Koderi et al., 2020) have reported moderately high activity (47.3% of MVIC) in the gluteus maximus during barbell RDL using seven repetitions and constant tempo while all our SLRDL conditions yielded substantially higher muscle activities in both SGM and IGM. Apart from the additional motor unit recruitment when performing high-speed actions, it is speculated that the use of unilateral load may have imposed extra demands on the gluteal contraction. In this regard, it is worth noting that all SLRDL conditions in our study have shown a higher activation in SGM than IGM. Ho et al. (2020) have addressed the unique fiber orientation of SGM for producing additional hip abduction (in the frontal plane) and external rotation (in the transverse plane) movements when compared with the function of IGM. Given the nature of single-leg standing and using unilateral resistance in SLRDL, it is believed that higher demand for stabilization tasks in the frontal and transverse plane is imposed on SGM.

Although ES-D and ES-ND were not the primary agonists for hip extension in SLRDLs, these muscles were highly active in our study to contract isometrically for spinal stabilization. In addition, most SLRDL variations in the current study yielded a moderate activation in EO-D and EO-ND while such a level of activation was believed to be sufficient to provide postural endurance and pelvic stabilization tasks (Hortobagyi et al., 2001), including antero-posterior tilt, lateral tilt, and transverse rotation. Besides, a high to very high activation in GM (40.6%–88.7%) was observed in our studies and these were comparable to the results demonstrated by Lane et al. (2019) and Stastny et al. (2016) using double-leg barbell and SL bodyweight RDL respectively. Since GM is an important stabilizer to control lateral pelvic tilt in the frontal plane, it is believed that the additional asymmetric load on SLRDL produced destabilizing torque on the pelvis in the frontal plane and led to a higher GM recruitment for pelvic stabilization. Based on these observations, unilaterally loaded SLRDL potentially provided dual training effects simultaneously including the strength enhancement of hip extensors and pelvic stabilization.

Interestingly, our results have shown no significant difference when comparing the FLY and DB SLRDL conditions. Only the non-clinical MBD showed a moderate increase of EO-ND when changing from DB-Ipsi to FLY-Ipsi conditions during concentric and also close to a moderate increase of EO-ND when changing the condition from DB-Con to FLY-Con during eccentric phases. Regarding the movement patterns of these two different SLRDL methods, both exercises required hip extension and posterior pelvic rotation in performing the concentric phase whereas hip flexion and anterior pelvic rotation of the supporting side were demanded during eccentric phase. Since the gripping position of dumbbell and the flywheel were standardized, the demand on maintaining spinal stability in both sagittal, frontal and transverse plane as well as the knee flexion angle of the supporting leg were comparable. Therefore, the identical movement patterns in the anatomical perspectives can justify for the high similarities of muscle activities between these two conditions. Nevertheless, it is noteworthy that the acceleration (during concentric) and deceleration (during eccentric) in DB SLRDL might induce certain inertia and probably slightly change the actual perceived loading whereas the FLY provided an isoinertial condition. In contrast, participants might not be able to anticipate the sudden change of loading between the concentric and eccentric phases in FLY conditions. All these factors might contribute to any observable activation differences between FLY and DB SLRDL exercises. Further studies focusing on the onset of muscle activations during different moments of FLY and DB SLRDL are warranted. Although the exact resistance torque applied on the body was supposed to be different between FLY and DB conditions, when the selected load of DB SLRDL was adjusted to accommodate for equivalent movement speed as the FLY SLRDL drills, both exercises produced comparable hip and trunk muscle activities. Theoretically, the flywheel could overload and hence produce higher muscle activities during eccentric action (Norrbrand, 2008). In fact, it seemed that balance might also negatively affect the activation of prime movers (Marchetti et al., 2016). In this regard, it is speculated that the decrease of the base of support in our SLRDL during FLY conditions as well as the sudden change of pulling direction when starting the eccentric phase might increase the instability and hence hinder the proposed benefits of additional activation of the agonists.

When comparing the muscle activities between loading positions in FLY and DB conditions, contralaterally loaded conditions of FLY-Con and DB-Con have shown significantly higher GM and SGM activities than those ipsilaterally loaded conditions of FLY-Ipsi and DB-Ipsi respectively. Given the unique but similar fiber orientations of these two gluteal parts for providing lateral pelvic stability in the frontal plane, both FLY-Con and DB-Con placed the load further away from the supporting leg as the axis of rotation and hence higher demands on lateral stability in both the trunk and pelvic region. Likewise, a similar observation was made in the ES-D muscle while the ES-ND has shown an opposite result (FLY-Con < FLY-Ipsi; DB-Con < DB-Ipsi). On the other hand, although the external oblique muscle was responsible for both rotational and lateral stability in trunk and pelvic regions, the SLRDL exercises provided the anti-flexion challenge to the spine rather than anti-extension, therefore EO-D and EO-ND were not as active as ES-D and ES-ND. Our results showed no significant difference between contralateral and ipsilateral conditions for EO-D and EO-ND parts whereas moderate effects existed when comparing the FLY-Ipsi or DB-Ipsi with FLY-Con and DB-Con. Such potential clear differences could be attributable to the additional anti-rotation works produced by EO for postural control and balance during SLRDL with different loading positions.

This study was not without limitations. The cross-talk might occur due to the very tight and close electrode placements and the overlapped abdominal or gluteal muscles. As SLRDL is a functional strength exercise highly challenging on neuromuscular control and balance, the determination of maximum strength using one or six repetition maximum was almost impossible. Therefore, the relative load to the corresponding maximum strength of each subject in performing SLRDL was not assessed.

## 5 Practical applications

The findings of the present study provide strength coaches and clinicians with empirical evidence for better exercise selection and implementation. Whenever strength coaches and clinicians look for using RDL exercise to strengthen the hip extensors, SLRDL using a dumbbell or flywheel with contralateral loading position is highly recommended to effectively strengthen the BF, SGM, IGM, GM, ES-D, and ES-ND muscles. Meanwhile, it also enhances the activation of trunk and pelvic stabilization muscles concurrently. Therefore, it can be a good option to potentially produce dual strengthening effects for both hip extensors and pelvic stabilizers. Given very high BF activation during the eccentric phase in FLY or DB conditions, SLRDL is potentially an effective method to enhance the eccentric strength for reducing the risk of hamstring tear. Coaches may also consider adding SLRDL drills into the warm-up routine to activate multiple trunk and hip muscles for better neuromuscular control before high-intensity training.

## Data Availability

The raw data supporting the conclusions of this article will be made available by the authors, without undue reservation.
